# Novel use of ink by territorial males in the coastal squid *Heterololigo bleekeri*

**DOI:** 10.1002/ecy.70576

**Published:** 2026-10-22

**Authors:** Shota Hosono, Shun Tokioka, Jun Yamamoto, Yoshio Masuda, Yuka Koike, Yoko Iwata

**Affiliations:** ^1^ Atmosphere and Ocean Research Institute The University of Tokyo Kashiwa Chiba Japan; ^2^ Shiogama Field Station, Fisheries Resources Institute Japan Fisheries Research and Education Agency Shiogama Miyagi Japan; ^3^ Field Science Center for Northern Biosphere Hokkaido University Hakodate Hokkaido Japan; ^4^ Miyagi Prefecture Fisheries Technology Institute Ishinomaki Miyagi Japan; ^5^ Present address: Ushimado Marine Institute Okayama University Setouchi Okayama Japan

**Keywords:** cephalopod, *Heterololigo bleekeri*, ink, reproductive behavior, squid, territory

Ink is a fascinating feature of cephalopods, primarily used for secondary defense in various forms. For instance, diffusive ink is used as a smokescreen that obscures a predator's view (Derby, 2014), or ink blobs suspended in the water are used as pseudomorphs that confuse predators (Hikidi et al., 2020). In addition to visual effects, chemical cues from ink have been suggested to deter predators from attack (Derby et al., 2013). Ink has also been proposed to serve as an alarm cue for conspecifics, both through visual (Wood et al., 2008) and chemical cues (Gilly & Lucero, 1992). Additionally, recent studies have revealed that ink is also used in contexts other than defense, such as predation (Sato et al., 2016), male–male competition (Allen et al., 2017), courtship (Nakayama et al., 2024), copulation (Sato et al., 2025), and spawning (Wada et al., 2006). Here, we report a novel use of ink by territorial males of the Japanese spear squid *Heterololigo bleekeri*, which was observed during captive observations of reproductive behaviors (Figure 1; Video S1). Our observations suggested that the released ink may be involved in territory maintenance against rival males and/or in attracting females to territories centered on a spawning substrate. As territoriality has been reported only in this species among squid (Tokioka et al., 2025), inking associated with territorial behavior is highly novel.

**FIGURE 1:**
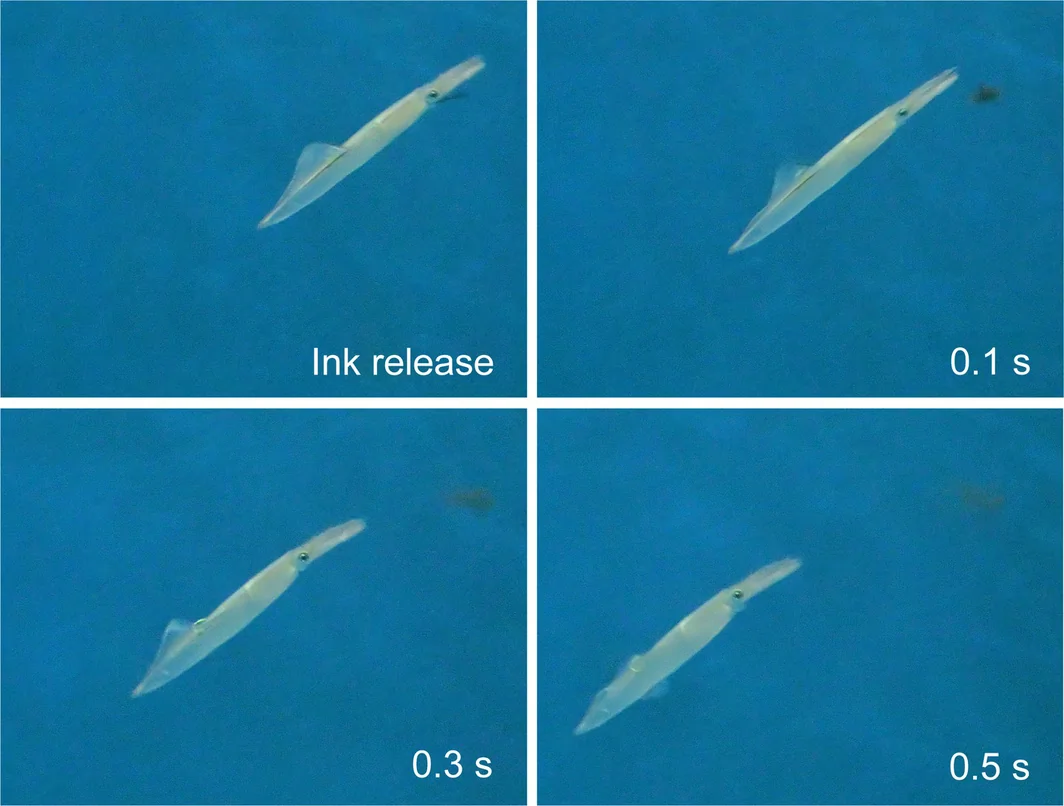
Sequence of inking behavior by a territorial male. The released ink was small and highly diffusive, dissipating in approximately 0.5 s. These images were cropped from Video S1, recorded with a digital camera (Nikon Z50 II) by Shota Hosono.

In March–April 2025, we observed a novel inking behavior in *H. bleekeri* during experiments examining their territoriality. The experiments were conducted in an experimental tank at the Hakodate Research Center for Fisheries and Oceans, Hokkaido, Japan. In the experiments, seven squid (five males and two females) and one spawning substrate were placed in the tank, and their behaviors were recorded using three fixed cameras. In addition to the fixed camera recordings, in‐person observations were conducted every 6 h for 2 h each (00:00–02:00, 06:00–08:00, 12:00–14:00, and 18:00–20:00) to collect data for defining territory establishment and loss (Tokioka et al., 2025). In this study, we analyzed three trials—Trials A, B, and C—lasting approximately 66, 55, and 44 h, respectively. In Trials B and C, the territory initially established by one male was taken over by another male during the trial. Observations continued after the takeover in Trial B, whereas Trial C was terminated after the takeover. Details of the experimental design are described in Appendix S1: Section S1 and Figure S1.

A total of 158 inking events were observed across the three trials (44 in Trial A, 66 in Trial B, and 48 in Trial C). Of these, 144 were performed by the territorial males while they were maintaining their territory (Figure 2A). Although territorial ownership changed during Trial B, the original territorial male released ink while holding the territory but stopped after losing ownership, whereas the male that subsequently acquired the territory began inking only thereafter. The remaining 14 inking events were performed by individuals that were not classified as territorial males based on our definition (Appendix S1: Section S2). Among the 144 inking events performed by territorial males, 14 occurred in rapid succession to another inking, within 2.0 s (Video S2). Since these successive ink releases occurred as a part of a single behavioral sequence (12 sequential events in total, including two cases of three successive ink releases), only the first inking from each sequence was used for further analysis. All ink observed in this study was small in amount and highly diffusive and dissipated within approximately 0.5 s (Figure 1; Videos S1 and S2). This type of ink is distinct from the highly viscous ink used for defense in this species (Appendix S1: Figure S2).

**FIGURE 2:**
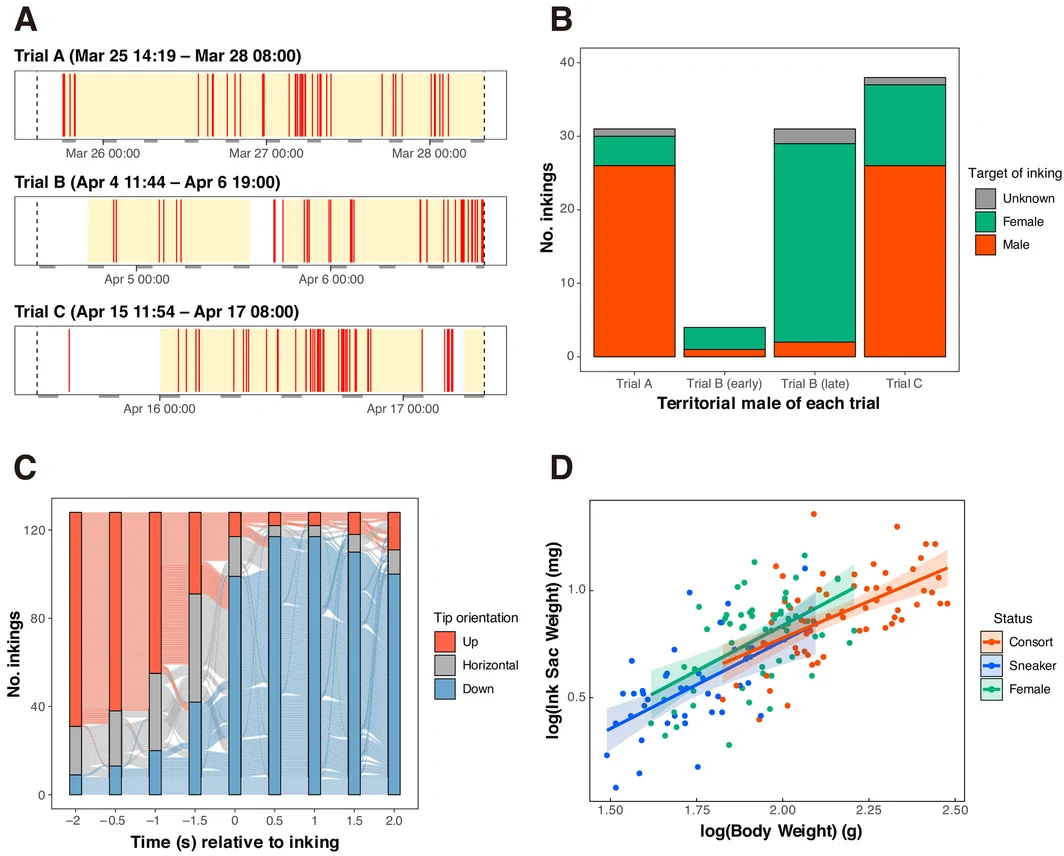
(A) Temporal data of the three trials. Red vertical lines indicate the timing of inking events, yellow shaded areas indicate defined territorial periods, and gray bars indicate periods of in‐person observation (00:00–02:00, 06:00–08:00, 12:00–14:00, and 18:00–20:00). (B) Number of inking events performed by territorial males during their territorial period. Colors represent the sex toward which territorial males directed their ink. (C) Temporal changes in mantle tip orientation around inking events (−2.0 to +2.0 s relative to inking). (D) Log‐transformed relationship between body mass and ink sac mass. Regression lines were estimated using ordinary least squares.

The remaining 130 inking events performed by territorial males occurred in one of three situations: an approach by the territorial male toward another individual (104/130), an approach by another male toward the territorial male (10/130), or no approach (16/130). When territorial males approached another individual, the sex of the target individual included both males and females, but the tendency to release ink toward either sex varied among territorial males (Figure 2B). Individuals targeted by territorial males did not exhibit obvious immediate behavioral responses. However, in two of all inking events performed by territorial males (*n* = 130), a female clearly followed a territorial male and entered the spawning substrate after the inking, one shown in Video S2. In addition, most inking events occurred on the side of the tank where the spawning substrate was placed (123/130), and inking events were observed under both bright (08:00–20:00, 77/130) and dark (20:00–08:00, 53/130) conditions.

To quantify the behavioral pattern of inking, we analyzed body posture from the videos every 0.5 s between −2.0 and + 2.0 s, with the moment of ink release defined as 0 s. Body posture at each time point was categorized as up, down, or horizontal based on the axis between the posterior mantle tip and the anterior mantle base (the head side). Of the 130 events, two were excluded from analysis due to being out of view for ±2.0 s during inking. The result showed a clear pattern of temporal change in tip orientation during the inking (Figure 2C). In 84.4% of inking events, the tip orientation was either up or horizontal at −1.0 s. However, from the moment of inking until 0.5–1.0 s afterward, the squid showed a tip‐down posture in 91.4% of inking events while swimming downward (Videos S1 and S2).

Males of *H. bleekeri* exhibit two distinct phenotypes associated with alternative reproductive tactics (consort/sneaker; Iwata & Sakurai, 2007), and territoriality is considered characteristic of consort males (Tokioka et al., 2025). To investigate the possible adaptations associated with territorial inking behavior, ink sac mass was compared among consorts (*n* = 65), sneakers (*n* = 43), and mature females (*n* = 60) using analysis of covariance (ANCOVA) with body mass as a covariate (detailed methods in Appendix S1: Section S3). No significant differences in ink sac mass were found between male tactics and sexes, with ink sac mass being explained only by body mass (Figure 2D; Appendix S1: Table S1).

In this study, we found that territorial males of *H. bleekeri* performed inking behavior while maintaining their territories. This finding suggests that the ink used in this context may contribute to the reproductive success of territorial males. Here, we propose two possible functions of the ink observed in this study. The first possibility is that ink functions as a signal advertising the competitiveness of territorial males to rival males. Because males of this species establish territories centered around a spawning substrate (Tokioka et al., 2025), territorial males have a higher chance of copulating with females right before spawning. Given that the last male to copulate with a female possibly achieves higher fertilization success in *H. bleekeri* (last‐male precedence: Iwata et al., 2005), maintaining a territory for visiting females is expected to enhance the reproductive success of males. Therefore, if the inking behavior is beneficial for territory maintenance by deterring rival males from intruding, it may consequently increase the reproductive success of territorial males. The second possibility is that ink functions as a signal advertising the attractiveness of territorial males and/or territories to females. Ink release by territorial males was also directed toward females, and in two cases, females appeared to respond by entering the spawning substrate. If females recognize the presence of high‐quality males or spawning substrates through a signal from the ink, inking behavior may increase mating opportunities and the reproductive success of territorial males. In addition, these two hypotheses are not mutually exclusive and may be valid simultaneously, depending on how the signal is interpreted by the receiver (competitiveness in males and attractiveness in females).

Released ink by territorial males was small in amount and highly diffusive, and inking events were observed under both bright and dark light conditions and even when territorial males did not approach other individuals, suggesting that the ink may function more as a chemical signal than as a visual signal, potentially acting as a pheromone. Chemical signals used for advertising territorial ownership are common in mammals and are generally known as scent marking (Ralls, 1971). Pheromone signals are also known to be used by aquatic animals to advertise social dominance to conspecifics (e.g., Barata et al., 2007). It is possible that chemical cues from the ink released by territorial males in *H. bleekeri* influence the behavior or physiological state of conspecifics and thereby contribute to their dominance. In addition, animals that use pheromones often perform specific behaviors, such as spraying urine (Uenoyama et al., 2024) or generating water currents (Kamio et al., 2008), to enhance chemical signaling. Given such behaviors, temporal changes in body axis orientation during inking by territorial males of *H. bleekeri* may facilitate chemical signaling by generating water flow that enables efficient dispersal of the ink.

Males of the cuttlefish species *Doratosepion andreanum* release ink during courtship and have larger ink sacs than the females (Nakayama et al., 2024). However, in *H. bleekeri*, such enlargement of the ink sac was not found in consort males compared with females or sneaker males, despite consort males establishing a territory and performing inking behavior. One possible explanation is that an enlarged ink sac may not be necessary because the amount of ink released during territory maintenance is very small.

This study suggests a novel animal strategy for territory maintenance through the inking behavior of the Japanese spear squid *H. bleekeri*. However, the function of the released ink remains unclear. One possible approach to test its function would be to introduce chemical cues from ink and examine changes in male–male competition or female preference for spawning substrates. Another approach would be to estimate the three‐dimensional positions of individuals and the spawning substrate to determine where and in which direction ink is released, as well as to examine in detail how other individuals respond after inking; however, such analyses were not possible in this study because the camera placement was not intended for position estimation. Future studies are expected to provide important insights into the effects of inking in territorial males and how ink use has been optimized and evolved in this species.

## AUTHOR CONTRIBUTIONS

Shota Hosono, Shun Tokioka, and Jun Yamamoto conducted the behavioral experiments. Yoshio Masuda collected the samples, and Shota Hosono and Yuka Koike measured them. Shota Hosono and Shun Tokioka performed all data analyses. Yoko Iwata supervised all experiments, and Shota Hosono and Yoko Iwata wrote the manuscript. All authors contributed critically to the drafts and approved the final version for submission.

## CONFLICT OF INTEREST STATEMENT

The authors declare no conflicts of interest.

## Supporting information


Appendix S1.

Video S1.

Video S2.

## Data Availability

Data (Hosono et al., 2026) are available in Dryad at https://doi.org/10.5061/dryad.kprr4xhkw.
