# Supplementary material for: Novel use of ink by territorial males in the coastal squid Heterololigo bleekeri
Source: Ecology. 2026 Oct 22;107(10):e70576. doi: 10.1002/ecy.70576 (PMC13687912; doi:10.1002/ecy.70576)
Supplement: Supplementary file 1 [file ECY-107-e70576-s001.pdf]

Appendix S1

Novel use of ink by territorial males in the coastal squid *Heterololigo bleekeri*

Shota Hosono, Shun Tokioka, Jun Yamamoto, Yoshio Masuda, Yuka Koike, Yoko Iwata.

Ecology

Section S1: Methods for the tank experiment

The inking behavior reported in this study was incidentally observed during experiments designed to investigate the territoriality of *Heterololigo bleekeri*. Therefore, the experimental design was not optimized for analyzing the inking behavior.

The experiments were conducted in an experimental tank (300 m^3; length 10 m, width 5 m, and depth 6 m; Figure S1A) at the Hakodate Research Center for Fisheries and Oceans, Hokkaido, Japan, in March–April 2025. The tank was filled with filtered seawater to a depth of 3 m, and the water temperature was maintained at 12°C using a water circulation system. Light conditions followed a 12:12 bright-dark cycle, with approximately 20 lx during the daytime (08:00–20:00) and approximately 8 lx during the nighttime (20:00–08:00), as measured by a lux meter attached to the spawning substrate (Figure S1B). The experimental tank had two acrylic windows, one at the front and one on the side. To record squid behavior, two fixed video cameras (Sony HDR-CX680 and FDR-AX45) were placed in front of the front window, and one fixed video camera (Sony HDR-CX430) was placed in front of the side window.

Squid used in these experiments were commercially caught by set nets and boat-operated lift nets in the Sea of Japan off Matsumae, Hokkaido, and Aomori Prefecture, Japan, and were transported alive by live-fish transport trucks to a commercial live-squid dealer. They were then transported to the center in a 250-L live-fish transport tank (Suiko, Japan) with oxygen supplied by bubbling. The sex of each squid was identified based on external observations, as the reproductive organs can be observed through the translucent mantle of live squid. Dissection following the experiments confirmed that all squid were mature and that all males were identified as consort males based on the morphology of their reproductive traits (Iwata & Sakurai, 2007).

Squid were maintained in stock tanks separately by sex until the start of a trial. Males were kept in a 20 m^3 circular tank and females in a 10 m^3 circular tank, in which filtered 12°C seawater was continuously circulated. In each trial, seven squid (five males and two females) and one spawning substrate were placed in the experimental tank. Before being introduced into the tank, squid were individually identified using ribbon tags on their fins. If a female died during a trial, it was replaced with another individual. The spawning substrate, made of plastic panels and pipes (1.1 m × 1.1 m × 1.3 m; Figure S1B; Tokioka et al., 2025), was placed on the bottom at a corner of the tank. Squid egg capsules spawned in a stock tank were artificially suspended from the inner ceiling of the spawning substrate to promote the establishment of a territory around it. In both the experimental and stock tanks, squid were fed sliced *Sardinops melanostictus* once a day at 08:00.

Video recordings were conducted throughout the duration of the trials. In addition, in-person observations were conducted every 6 h for 2 h each (00:00–02:00, 06:00–08:00, 12:00–14:00, and 18:00–20:00). During these observations, agonistic behaviors between males were monitored continuously, and the nearest individual to the spawning substrate was recorded every 5 min. Using the data from in-person observations, territory establishment and loss were defined following Tokioka et al. (2025). Therefore, the true territory periods do not always coincide with defined territorial periods. For example, if territory establishment was identified during the 18:00–20:00 in-person observation, the territory was defined as having been established at 18:00, even though it may have been established as early as 16:00. Similarly, if territory loss was identified during the 18:00–20:00 in-person observation, the territory was defined as having persisted until the preceding observation (14:00), even though it may have continued until 17:00. Each trial was ended when more than two males died during the trial or when sufficient data were collected for

the original experiment. In this study, videos of three trials (A, B, and C) were analyzed for inking behavior.

The experimental procedures were approved by the Life Science Research Ethics and Safety Committee of the University of Tokyo (P24-2).

Section S2: Supplementary results for inking

In this study, we observed 158 inkings across three trials. Of these, 14 cases were performed by individuals that were not classified as territorial males at the time of inking according to the territory definition based on in-person observations. However, 12 of these 14 cases were performed by individuals that were classified as territorial males either from the following in-person observation onward (4 inkings in Trial B during 14:00–18:00 Apr. 5; Figure 2A) or until the preceding in-person observation (8 inkings in Trial C during 02:00–06:00 Apr. 17; Figure 2A). Thus, although these individuals were not categorized as territorial males according to our definition (see Section S1), they were likely behaving as territorial males at the time of inking. The remaining two inkings were considered to have been performed by genuinely non-territorial males: one occurred when another individual possessed a territory (Trial B, Apr. 5 at 04:58), and the other occurred before any territory had been established (Trial C, Apr. 15 at 15:04). These two inkings were performed by different individuals, neither of which established a territory during the trial. The ink released in these two events was also small in amount and highly diffusive. In this study, no inkings by females were observed.

Section S3: Methods for morphological analysis

Squid samples were commercially fished by bottom trawls and set nets off Sendai Bay, Miyagi Prefecture, Japan, from January to May 2024, and transported on ice to the Atmosphere and Ocean Research Institute. In the laboratory, each squid was measured for body weight to the nearest 0.1 g, and its sex and maturity were determined by examining the reproductive organs through dissection. In mature males, the reproductive tactic of each individual was identified based on the morphology of spermatophores and spermatangia, following Iwata & Sakurai (2007). For each individual, the ink sac was removed and rinsed with water to remove the contained ink. The empty ink sac was then placed on Petri dish and dried at 60°C for 12 h. Finally, the dried ink sac weight was measured to the nearest 0.0001 g for 65 consorts, 43 sneakers, and 60 mature females. To compare ink sac weight among reproductive statuses (i.e., consort, sneaker, and mature female), an analysis of covariance (ANCOVA) with log(ink sac weight) as the dependent variable and log(body weight) and reproductive status as predictors was performed. Model comparison based on AIC favored the model without the interaction between log(body weight) and reproductive status, and the best model indicated that the slopes did not differ among reproductive statuses. Statistical analyses were conducted using R 4.4.2.

Figure S1

A

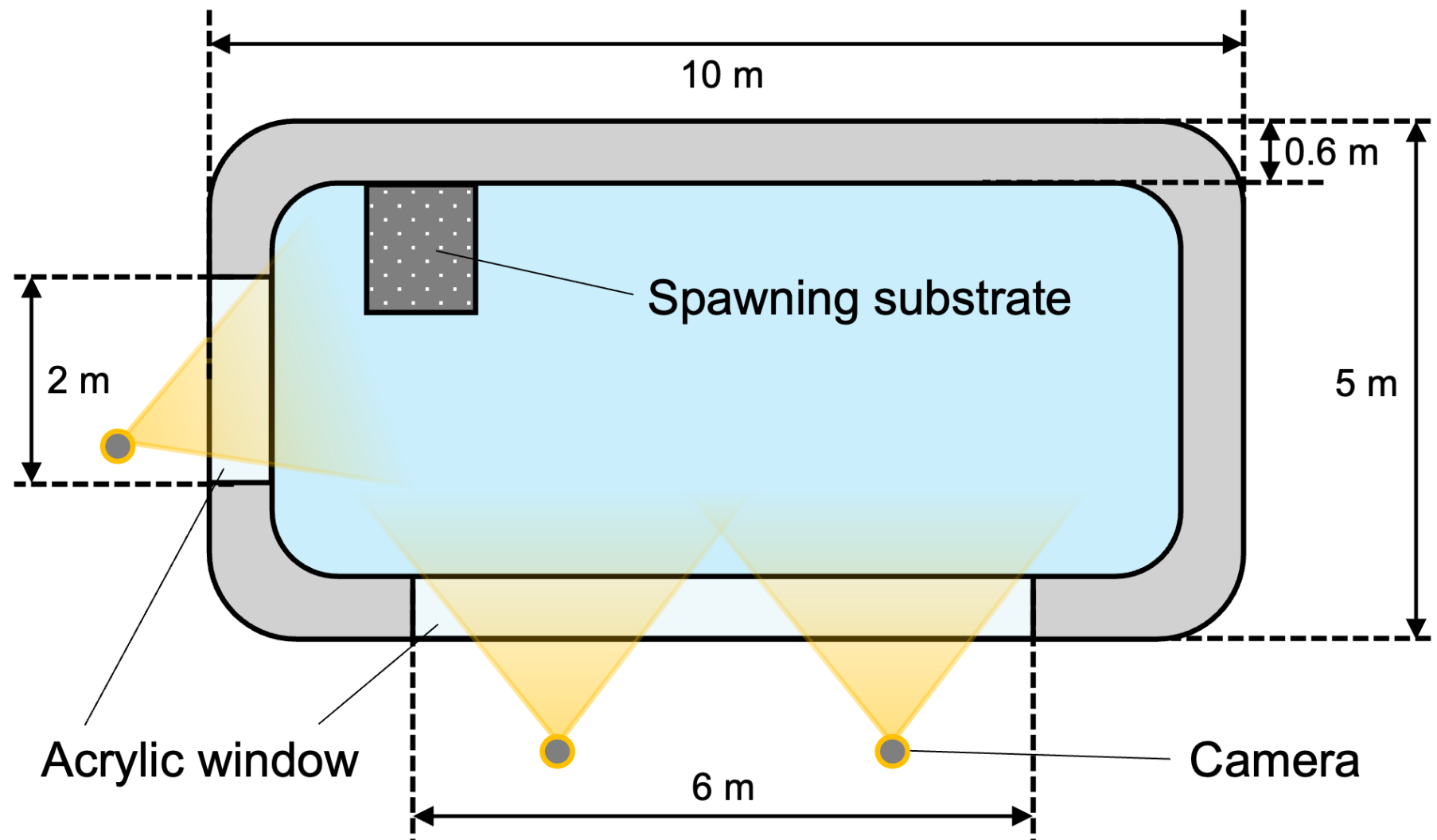

B

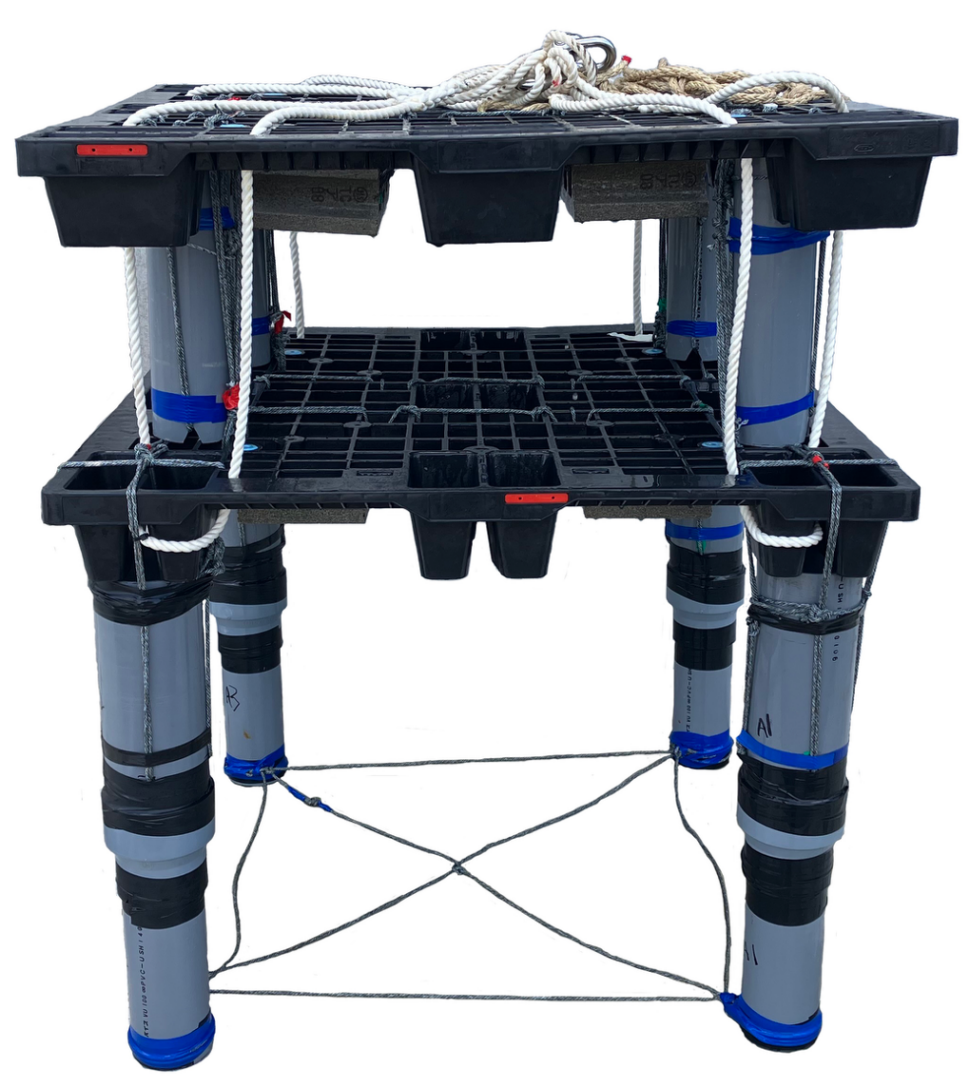

Figure S1. (A) Top view of the experimental tank. (B) Artificial spawning substrate used in the experiment. Female squid deposited egg capsules on the underside of the inner ceiling, and males established territories centered around the substrate. The photograph was taken by Shun Tokioka and edited by Shota Hosono to remove the background.

Figure S2

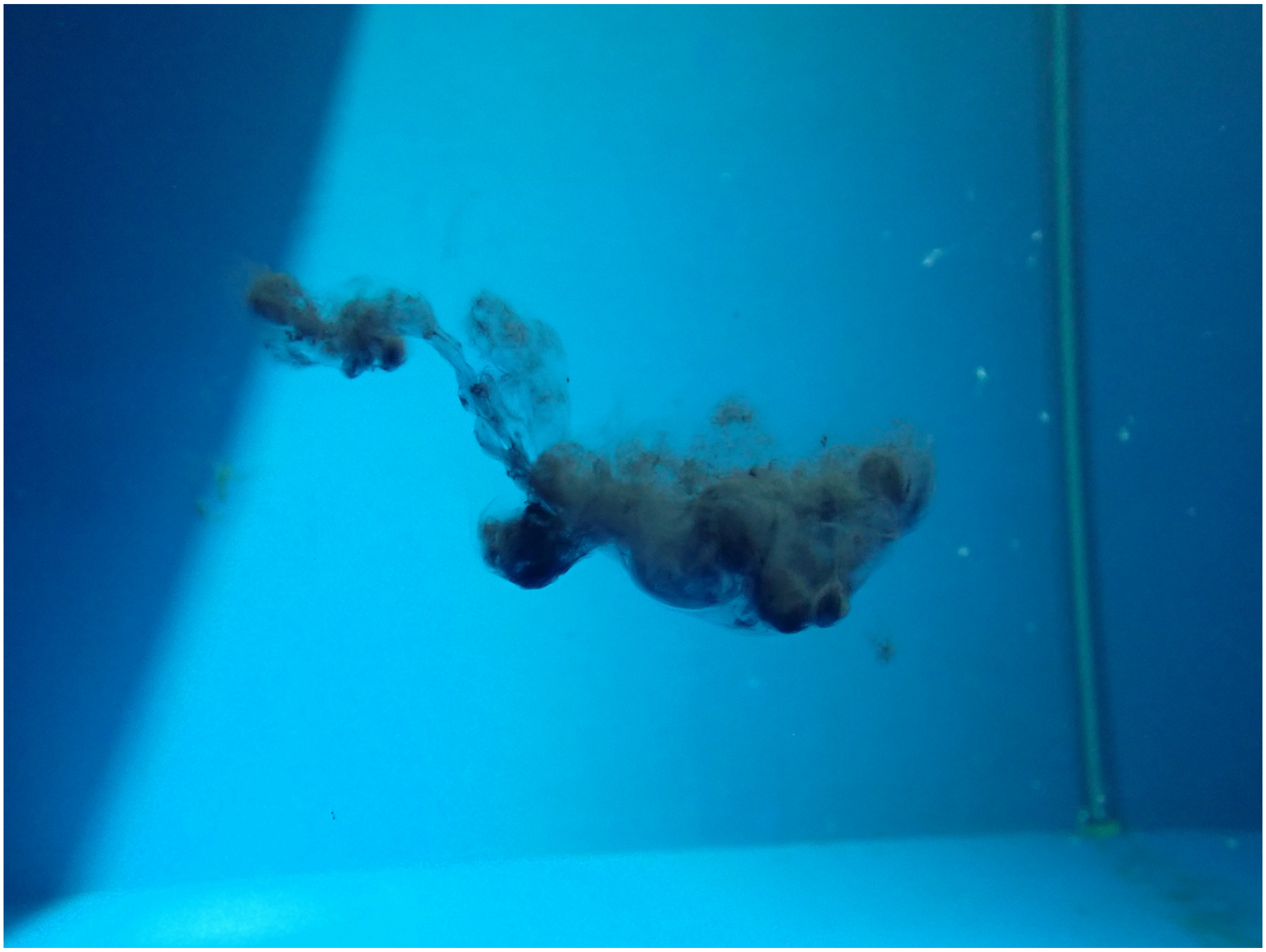

Figure S2. Highly viscous ink released by *Heterololigo bleekeri*. This ink was released when the squid was chased with a net during capture from the tank after an experiment, suggesting that it is associated with defense. The ink remained suspended in the water for some time without diffusing. Photograph taken by Shota Hosono using a digital camera (Nikon Z50 II).

Table S1. Results of ANCOVA with log(ink sac weight) as the dependent variable and log(body weight) and reproductive status (i.e., consort, sneaker, and mature female) as the predictors.

Predictor	Estimate	SE	t-value	p-value
(Intercept)	-0.76	0.19	-3.98	**< 0.01**
log(body weight)	0.76	0.09	8.69	**< 0.01**
Status (mature female)	0.06	0.04	1.73	0.09
Status (sneaker)	-0.02	0.05	-0.31	0.76

References

Iwata, Y. and Y. Sakurai. 2007. “Threshold Dimorphism in Ejaculate Characteristics in the Squid *Loligo bleekeri*.” *Marine Ecology Progress Series* 345: 141–146.

Tokioka, S., J. Yamamoto, J. R. Bower, H. Matsui, S. Suzuki, and Y. Sakurai. 2025. “First Quantitative Evidence of Territorial Behavior in Squid Near Spawning Substrates.” *Scientific Reports* 15: 29061.
